# Investigation of eye movement characteristics during free throws at varying intensities among basketball players and its correlation with free throw percentage

**DOI:** 10.1371/journal.pone.0299938

**Published:** 2024-08-22

**Authors:** Chunzhou Zhao, Na Liu, Sunnan Li, Xuetong Zhao

**Affiliations:** 1 Beijing Normal University-College of P.E and Sports, Beijing, China; 2 Guangdong Country Garden Polytechnic, Qingyuan, China; Erzurum Technical University: Erzurum Teknik Universitesi, TÜRKIYE

## Abstract

**Background:**

Free throws serve as a crucial scoring mechanism in basketball games. As the level of basketball and the intensity of competition continue to improve, the frequency of free throw attempts gradually rises. However, with heightened game intensity, maintaining a consistent free throw success rate becomes increasingly challenging. The aim of this study was to investigate the eye movement characteristics exhibited by basketball players during free throws of varying intensities, as well as explore the relationship between these eye movement characteristics and free throw percentage.

**Methods:**

Twenty elite female basketball players were recruited to perform free throws at varying exercise intensities (low, moderate, and high) while wearing an eye tracker device. Eye tracking data was collected using the Tobii Glasses 3 eye tracker. Additionally, the Polar team pro, a heart rate monitoring system manufactured in Finland, was utilized to monitor participants’ heart rates during different exercise intensities.

**Results:**

The average number of fixations on the hoop and net during free throws of varying intensities exhibited statistically significant differences (P < 0.05). The fixation durations of the hoop, backboard, and net exhibited statistically significant differences (P < 0.05). Notably, the hoop accounted for the highest proportion of the overall average fixation duration. high-intensity free throws necessitate lengthier processing durations compared to low-intensity and medium-intensity free throws. Furthermore, for free throws of moderate intensity, there was a significant negative correlation between the number of fixations on the hoop and free throw percentage (P < 0.05). Conversely, for high intensity free throws, there was a significant positive correlation between the fixation duration on the hoop and free throw percentage (P < 0.05).

**Conclusions:**

Under the three kinds of sports intensity, the players mainly focus on the hoop position, the moderate intensity free throw has the best stability, and the information search strategy and information processing efficiency are the highest. In high intensity free throws, the longer the fixation duration at the hoop, the higher the free throw percentage; The higher the number of fixations at the hoop, the lower the percentage of free throws.

## Introduction

Eye tracking technology, as a real-time and effective method for investigating cognitive processing in individuals, is a technology that quantifies fixation, saccadic movements, and saccades based on visual input. It provides us with empirical data resources through the utilization of eye tracker [1]. In recent years, there has been an increasing research interest in the application of eye movement technology within the sports domain [2–8]. In the domain of basketball, it is primarily employed to analyze participants’ fixation count, duration, and scanning trajectory during visual search within the area of interest. This facilitates identification of participants’ fixation characteristics and provides a foundation for basketball instruction and training. Free throws are a complex aiming skill that necessitates the integration of visual information acquired through apparent eye movement and the execution of precise aiming movements [9]. The quality of a shot primarily relies on shooting mechanics, head and eye positioning (as direct fixation at the target significantly enhances shot accuracy), and attentional focus (since direct gaze at the target optimizes shot accuracy) [10].

Attention plays a pivotal role in motor learning and performance [11]. Attention training can significantly enhance shooting accuracy among basketball players [12], with particular emphasis on self-paced [13] and closed-skill tasks [14] Such as accurate basketball free throws. Attention is considered to regulate the detection of information [15], and the allocation of external attention significantly impacts optimal performance [16, 17]. Skilled players adeptly shift their gaze in response to the shooter’s movements, aiming to extract crucial visual cues that may forecast a successful shot [18]. Basketball players exhibit prolonged durations of quiet eyes during free throw execution, and superior performance is associated with reduced fixations [19]. Gong Ran et al. discovered that basketball players possess the ability to anticipate future target trends compared to the general population, and their adaptable fixation strategy provides compelling evidence for the coexistence of visual indexing and multifocal attention theory [6]. With the rapid advancement of basketball proficiency, the on-court competition has escalated in both the American Basketball League and the Chinese Basketball League. Approximately 50 free throws are attempted per game, with instances where the success rate of these shots determines the outcome [20].

With the intensification of competition, there is a corresponding increase in free throw opportunities, particularly during close-score situations where the decisive free throw at the final moment often determines the game’s outcome. Consequently, teams are placing greater emphasis on training and enhancing their free throw techniques. Liu’s research indicates that free throws typically contribute to 15–20% of total points scored in a game; however, in certain instances, they can account for as much as 40–60 points [21]. Elite athletes possess profound basketball knowledge, honed specialized skills, and extensive experiential accumulation. During the free throw process, they employ efficient visual search to make decisions, meticulously process the gathered information, and utilize adept motor skills to execute successful shots. Attention plays a pivotal role in basketball shooting as it serves as a prerequisite for developing optimal muscle proprioception and enhancing shooting accuracy [22].

Currently, the predominant approach in basketball research utilizing eye trackers involves assessing participants’ eye movement patterns through the presentation of images or videos on a screen. While this method ensures accuracy in data collection, there remains a discernible gap between experimental settings and real-game scenarios in terms of ecological validity. Basketball is a physically demanding competitive sport primarily reliant on physical strength. Endurance, particularly specialized endurance, plays a pivotal role in achieving long-term performance excellence. The intensity of the load exerted during training and competition is crucial for optimal outcomes, encompassing both maximum and sub-maximum levels [23]. The fundamental essence of load intensity lies in its close association with the intensity of competition [23]. Enhancing athletes’ competitive performance ability under specific training load intensities holds practical significance solely based on the characteristics of competition load. According to Zhang’s study, high-intensity basketball matches account for 41%, medium-intensity matches account for 44%, and low-intensity matches account for 15%. The ratio of high, moderate, and low intensity is reported as 2.7:3:1 [24]. Basketball players can maintain a high shooting accuracy during regular training or low-intensity free throws; however, it becomes challenging to sustain this high percentage under increased exercise intensity during the game, particularly in the latter half. Successful free throws require narrow external focus and internal awareness of bodily sensations while being responsive to various physical cues. Any distractions that divert attention from these factors will inevitably lead to reduced free throw success rates [25]. From the perspective of limited cognitive resources, the decline in motor performance primarily stems from excessive occupation of cognitive resources by task-irrelevant information, resulting in insufficient allocation of cognitive resources to the target task and thereby impeding focused attention on task-related information processing. Specifically, the decline in motor performance is frequently accompanied by a concomitant decrease in attentional control, an augmented focus on irrelevant or threatening information, a reduced duration of attention towards the target, and an increased difficulty in disengaging attention from distracting information [26].

In regular training, coaches often solely prioritize the skills and physical conditioning of players during free throw practice sessions. During a basketball match, as exercise intensity escalates, maintaining a high free throw percentage becomes increasingly challenging for players. Currently, there is a lack of clarity regarding the alterations in athletes’ eye movement index when shooting free throws at varying intensities, which has not garnered sufficient attention. Additionally, the relationship between the eye movement index and free throws percentage at different intensities remains unclear. Building upon previous research, this study employs advanced instrumentation to investigate the eye movement characteristics of elite basketball players during free throw shooting at varying intensities and explores the relationship between these characteristics and free throw percentage. The findings aim to provide scientific guidance for psychological training, teaching, and competition in basketball free throws.

## Methods

### Participants

The present study recruited a cohort of twenty participants from the female basketball school team at Beijing Normal University. All participants were distinguished as champions of the Chinese University Basketball Association (CUBA) in 2021, with five individuals additionally holding titles as champions of the women’s basketball team at the 2023 World University Games. The participants’ age range was 18–24 years (mean: 21.56; SD: 2.47 years), with an average of more than 10 years of experience per participant (mean: 10.09; SD: 1.96) and a mean weekly training duration of over 15 hours (mean: 15.02; SD: 2.86 hours) in the past year. The participants were required to have a minimum free throw percentage of at least 65%. Additionally, it should be noted that all participants exhibited right-handed shooting proficiency. All participants actively engaged in the experiment and were duly compensated for their valuable time. The study obtained approval from the Ethics Committee of Beijing Normal University-College of Physical Education and Sports. (No. 20221126). The experiment commenced only after all participants had provided written informed consent. Furthermore, it was ensured that all participants possessed a dominant right hand for free throws and exhibited normal visual acuity.

### Apparatus

The Tobil Glasses 3 eye tracker was utilized to capture the participants’ free throw videos, with a sampling frequency of 100Hz. The eye movement data were collected using Tobii Pro Lab (version 1.21.21571) software, while the participants’ heart rate at different exercise intensities was monitored using a Polar Team Pro device from Finland.

### Exercise intensity division

According to the standard of human body’s response to exercise load and heart rate, it is generally categorized into four levels of intensity: high intensity, defined as a heart rate exceeding 157 beats per minute; moderate intensity, ranging from 156 to 139 beats per minute; low intensity, ranging from 138 to 120 beats per minute; and general activities, characterized by a heart rate below 120 beats per minute [24]. In accordance with the exercise intensity grading standard proposed by Chen et al [27] and considering the actual intensity observed in basketball matches, the exercise intensity of free throws was categorized into three levels: low intensity (120–138 times/min), moderate intensity (139–156 times/min), and high intensity (157 times/min).

### Experimental scenarios

The experiment was conducted at the basketball court of Beijing Normal University Gymnasium. During different exercise intensities (low, moderate, and high), participants wore an eye tracker while performing free throws. Each participant completed three sets of free throws at varying intensities, with data being recorded.

### Design of experiment

The present study employed a within-group design. The independent variable, exercise intensity, was operationalized into three levels (low, moderate, and high). The dependent variables consisted of eye movement indices, specifically the number of fixations and fixation duration.

### Experimental procedure

The experiment was concluded on December 25, 2022, and the entire experiment was completed under the guidance of the experimenter. Prior to commencing the experiment, all participants were provided with a comprehensive explanation of the theoretical background, experimental requirements, and necessary precautions. Simultaneously, all participants were equipped with heart rate sensors, and subsequently, based on the draw order, a participant entered the basketball court to enhance exercise intensity through activities such as running back, dribbling, and shooting during the march, this approach was also employed when participants transitioned from low intensity to both moderate and high intensity levels. The Polar Team Pro was utilized for monitoring participants’ heart rates. Meanwhile, other participants waited in the break room. Prior to the experiment, the experimenter instructed the participants to adhere to a predetermined warm-up protocol in order to prepare for the upcoming study. Subsequently, when the participants’ heart rate reached the predetermined exercise intensity, they positioned themselves at the free throw line and commenced the test in accordance with the regulations governing free throws. Each participant attempted three free throws under three different exercise intensities. The initial attempt was utilized for data analysis purposes, while the subsequent two attempts served as backups. All participants underwent a low-intensity free throw test in the initial round, a moderate-intensity free throw test in the subsequent round, and a high-intensity free throw test in the final round. The average duration of testing for each participant was approximately 20 minutes, with the entire experiment spanning around 400 minutes.

### Free throw percentage test

The free throw percentage was tested at the same location and using identical experimental procedures. In the afternoon, the testing methods and times remained consistent with those employed in the morning session. The assessment spanned five days, during which 100 free throws were tested under each exercise intensity level. The successes and failures of free throws were meticulously recorded to ultimately calculate the participants’ free throw percentages for each exercise intensity.

### Area of interest (AOI)

**Fig 1:**
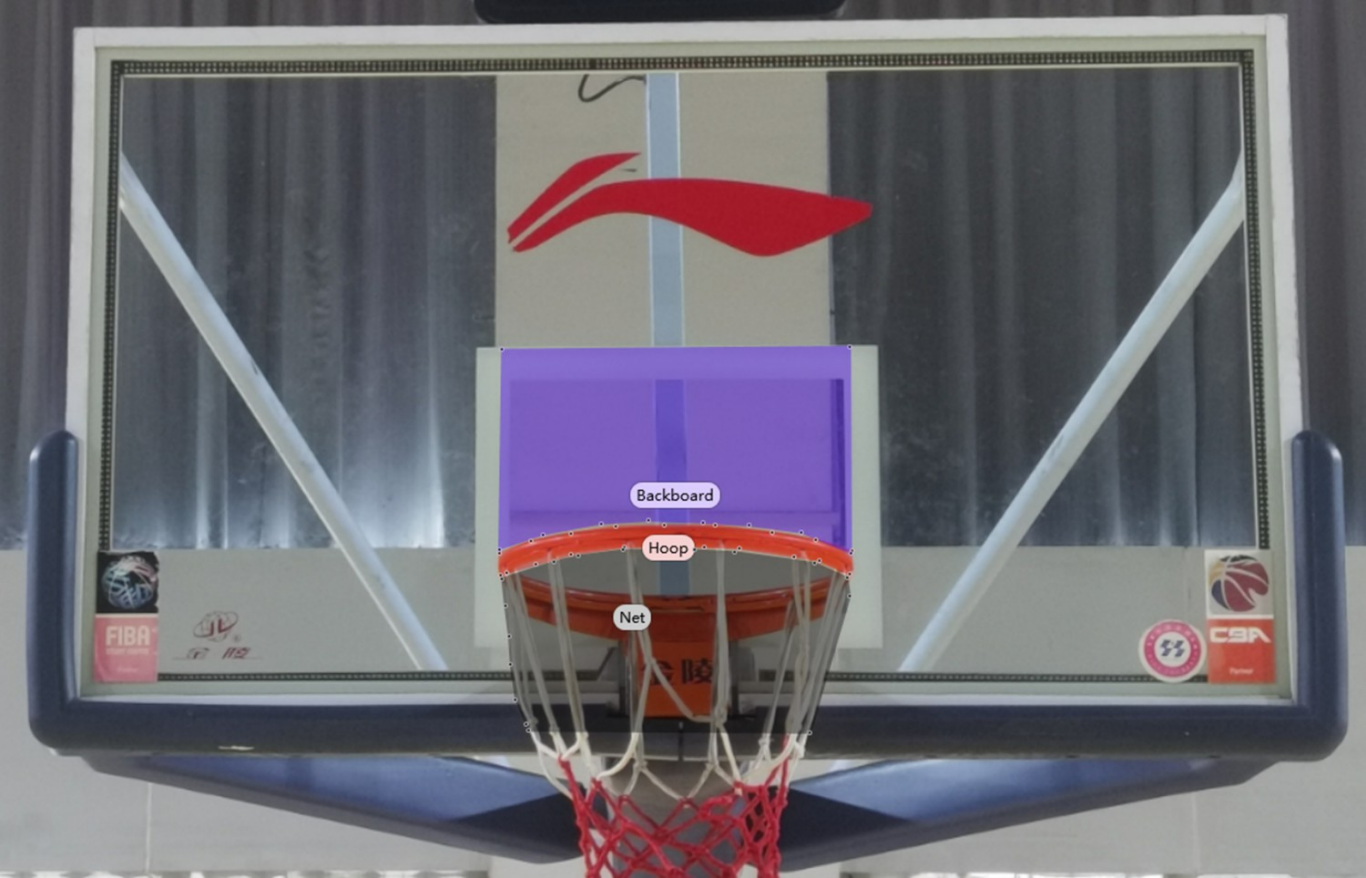
Area of interest (AOI) based on free-throw fixation position.

The area of interest refers to the specific area that participants fixation on during the free throw. By collaborating with basketball experts and instrument manufacturers, and considering the gaze position of participants throughout the free throw, we categorized the region of interest into three distinct areas. see Fig 1.

### Ethics statement

This study was conducted in accordance with the recommendations of the Ethics Committee of the College of Physical Education and Exercise, Beijing Normal University, and written informed consent was obtained from all participants. Participants gave written informed consent in accordance with the Declaration of Helsinki. The experimental protocol was approved by the Ethics Committee of Beijing Normal University (No.20221126).

### Dependent variables and data analysis

#### Eye tracking data

Fixation: A fixation was defined as a condition in which the eye remained stationary within a tolerance of movements up to 3° for a period greater than100ms [28]. Fixation duration: The total duration of the fixations inside this area of interest during an interval (Tobii Pro lab User Manual). Fixation count: The number of fixations occurring in this area of interest during an interval (Tobii Pro lab User Manual).

#### Data analysis

The data collected from the free-throw video encompassed the duration starting from when the participants initiated their fixation duration until after the basketball was released. After editing this video, Tobii Pro Lab (version 1.21.21571) was employed to analyze participants’ eye movement data during the execution of free throws. The collected data were entered into SPSS 26.0 in the form of an Excel table for statistical analysis and ANOVA presentation purposes. In case of a significant effect, the Least Significant Difference (LSD) test was employed for conducting multiple comparisons. Furthermore, a Pearson correlation test was conducted to examine the relationship between the number of fixations, fixation duration, and hit rate. The standard level of significance was set at p<0.05.

## Result

### Number of fixations

In order to investigate the disparity in the mean number of fixations among participants on each AOI during free throws under varying exercise intensities, a variance analysis was conducted with exercise intensity as the independent variable and the number of fixations on each AOI as the dependent variable (refer to Table 1). Following K-S test, it was determined that the number of fixations by all participants during free throws under the three exercise intensities exhibited a normal distribution.

**Table 1:** Comparison of number of fixations across different AOI at varying intensities(times).

	Low intensity		Moderate intensity		High Intensity			
AOI	M	SD	M	SD	M	SD	F	P value
Hoop	1.60	0.60	1.60	0.50	2.10	0.72	4.469	0.016^*^
Backboard	1.35	0.50	1.30	0.47	1.60	0.60	1.849	0.160
Net	1.20	0.41	1.05	0.22	1.60	0.60	8.416	0.001^*^

^*^The mean difference is significant at the 0.05 level.

The results presented in Table 1 demonstrate a significant disparity in the average number of fixations of the hoop across the three exercise intensities (F _(2, 57)_ = 4.469, P = 0.016, η2 = 0.135). Subsequent SLD multiple comparisons revealed a noteworthy distinction in the average number of fixations between low and high intensity (P < 0.05). Additionally, there was a significant discrepancy observed in the average number of fixations between moderate and high intensity exercises (P < 0.05). The average number of fixations of the nets exhibited a significant difference among the three exercise intensities (F _(2, 57)_ = 8.146, P = 0.001, η2 = 0.228). Subsequent SLD multiple comparisons revealed a significant disparity in the average number of fixations between low intensity and high intensity (P < 0.05). Moreover, there was a notable distinction in the average number of fixations between moderate intensity and high-intensity exercises (P < 0.05).

### Fixation duration

In order to investigate the disparity in average fixation duration among participants within each AOI during free throws under varying exercise intensities, a variance analysis was conducted with exercise intensity as the independent variable and fixation duration of each AOI as the dependent variable (refer to Table 1). Following the K-S test, it was determined that fixation durations of all participants during free throws under the three exercise intensities exhibited normal distribution.

The results presented in Table 2 demonstrate a significant disparity in the average fixation duration of the hoop across the three exercise intensities (F _(2, 57)_ = 12.15, P = 0.000, η2 = 0.311). Subsequent LSD multiple comparisons revealed that there was a statistically significant difference between the average fixation duration of low intensity and high intensity exercises (P < 0.05), as well as between moderate intensity and high intensity exercises (P < 0.05). The average fixation duration of the backboard exhibited a significant difference, as indicated by F _(2, 57)_ = 6.785, P = 0.002, η2 = 0.192. Subsequent LSD multiple comparisons revealed significant disparities between low intensity and moderate intensity (P < 0.05), as well as between low intensity and high intensity (P < 0.05). Additionally, there was a noteworthy distinction observed between moderate intensity and high intensity (P < 0.05).

**Table 2:** Comparison of fixation duration across different AOI under varying intensities (Ms).

	Low intensity		Moderate intensity		High Intensity				
AOI	M	SD	M	SD	M	SD		F	P value
Hoop	1014	91	1029	74	911	73		12.851	0.000^* *^
Backboard	703	106	635	60	818	54		6.785	0.002^*^
Net	387	79	288	58	494	53		51.258	0.000^* *^

^*^The mean difference is significant at the 0.05 level.

^* *^The mean difference is significant at the 0.01 level.

The average fixation duration of the net differed significantly, as indicated by F _(2, 57)_ = 51.2585, P = 0.000, η2 = 0.643. Further LSD multiple comparisons revealed significant differences between low intensity and high intensity (P < 0.05), moderation intensity and high intensity (P < 0.05), as well as low intensity and moderate intensity (P < 0.05).

### The distribution characteristics of number of fixations and fixation duration

**Fig 2:**
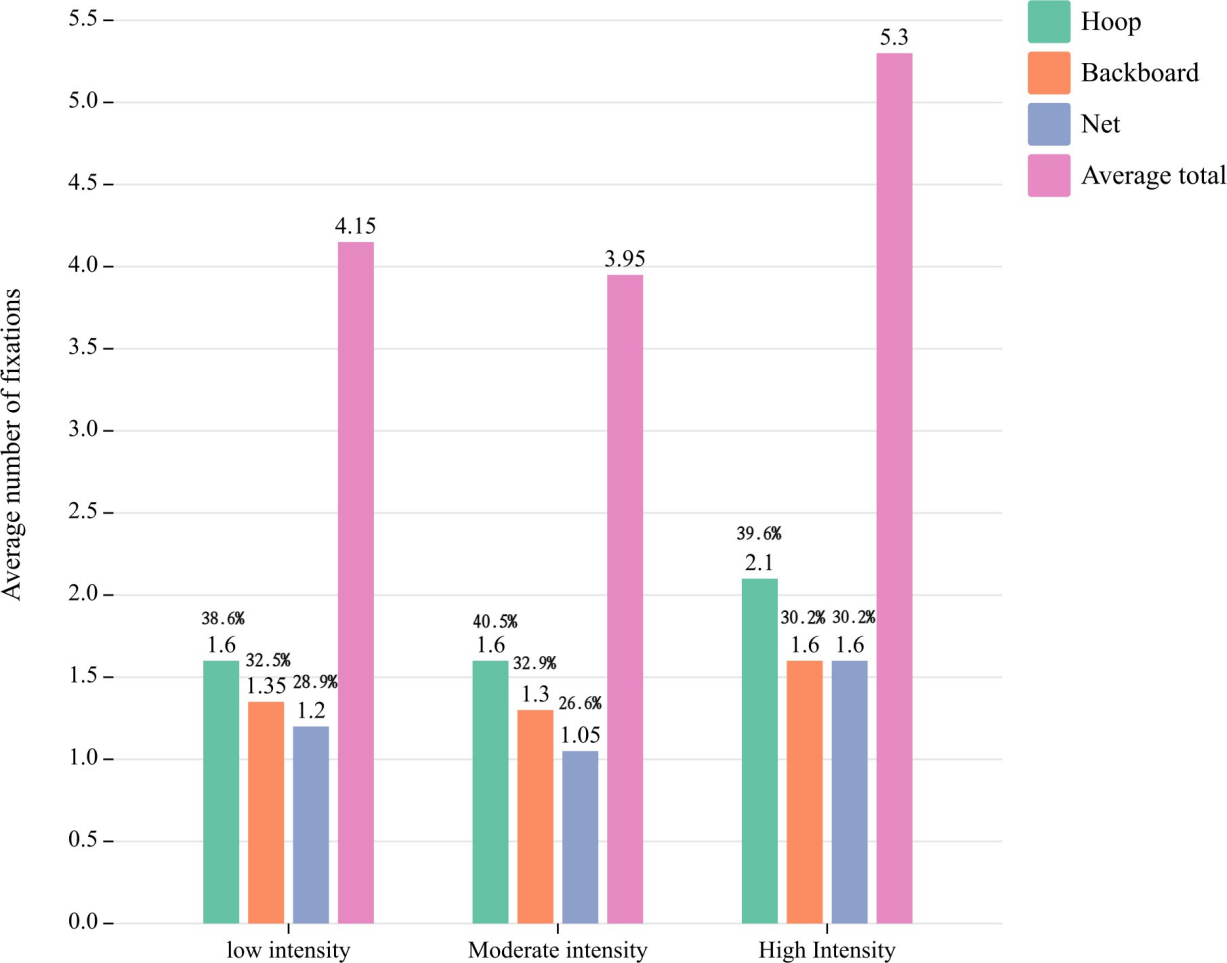
The distribution of number of fixations.

The results depicted in Fig 2 indicate that the moderate intensity free throw elicited the lowest average total number of fixations, while the high intensity free throw resulted in the highest average total number of fixations. The average number of fixations of the hoop in the three exercise intensities constituted the largest proportion of the total average number of fixations, with percentages of 38.6% at low intensity, 40.5% at moderate intensity, and 39.6% at high intensity, respectively. These findings suggest that players primarily focused their attention on the hoop during free throw. The average proportion of net fixations in relation to the total average number of fixations was found to be significantly lower for low and medium intensity free throws. Additionally, the average number of backboard and net fixations was observed to be equivalent to the average number of net fixations for high intensity free throws, suggesting that variations in intensity exerted an influence on athletes’ fixation patterns during free throw attempts. For low and moderate intensity free throws, the average number of fixations on the net constituted the smallest proportion of the overall average number of fixations. For high intensity free throws, both backboard and net had an equal average number of fixations (30.2%), indicating that exercise intensity influenced players’ number of fixations.

**Fig 3:**
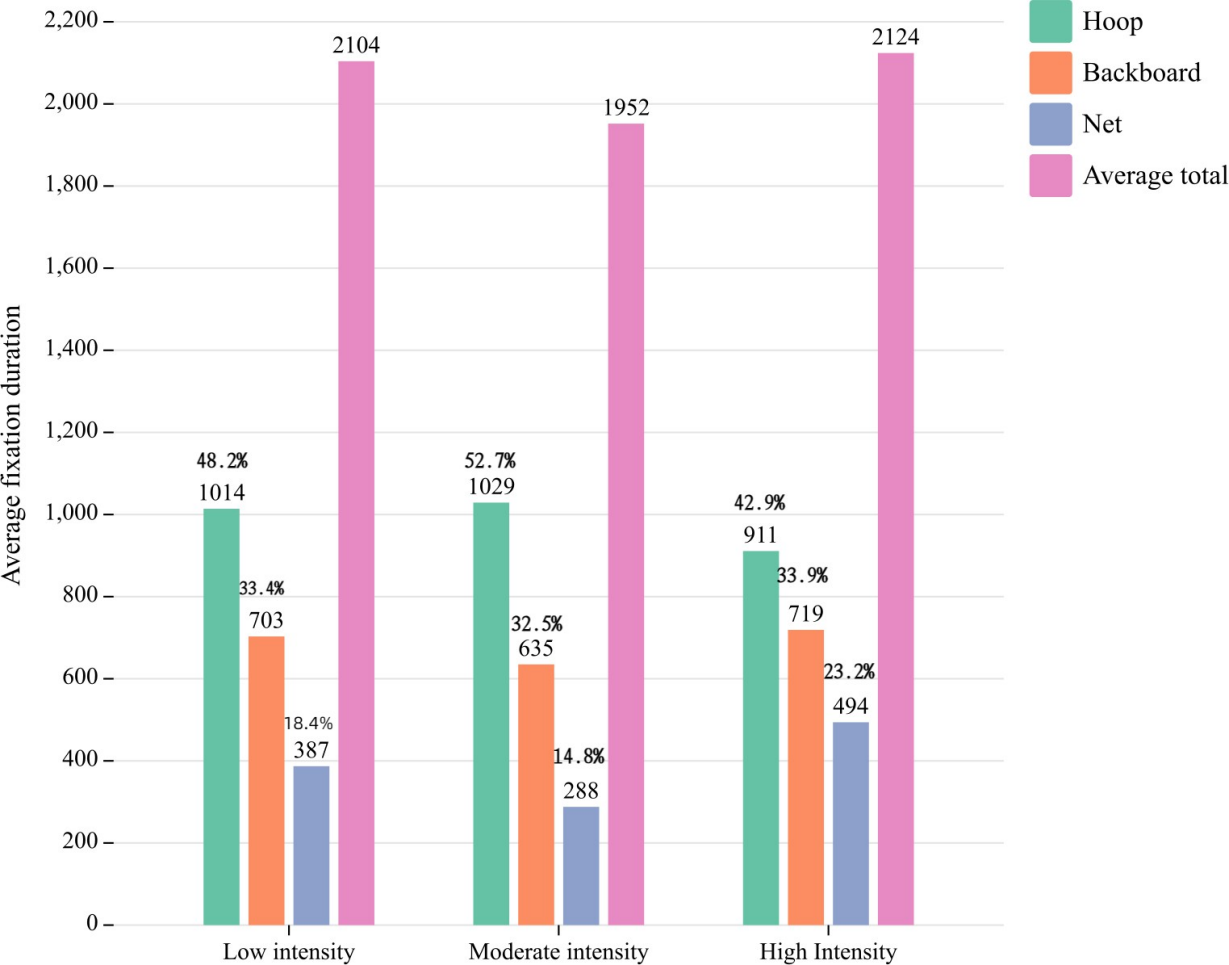
The distribution of fixation duration.

The results depicted in Fig 3 demonstrate that the total average fixation duration was significantly shorter for the moderate intensity free throw (1952 ms), followed by the low intensity condition (2104 ms), and reached its maximum duration during the high intensity free throw (2124 ms). The average fixation time on the hoop constituted the largest proportion of the total average fixation duration for the three intensity levels of free throws, accounting for 48.2% at low intensity, 52.7% at moderate intensity, and 42.9% at high intensity. The average fixation duration of the net was the shortest, constituting the smallest proportion of the total average fixation duration. However, in the low-intensity and high-intensity free throw, the average fixation duration of the nets increased to 18.4% and 23.2%, respectively. The aforementioned findings suggest that the exercise intensity exerts an influence on the fixation duration during free throw.

### The correlation between the number of fixations and the success rate of free throws

The Pearson correlation coefficients were computed for the number of fixations, fixation duration, and free throw percentage. The magnitude of each correlation coefficient was assessed using the following classification criteria: < 0.1 = negligible; 0.1–0.3 = weak; 0.3–0.5 = moderate; and 0.5–0.7 = strong [29].

As depicted in Table 3, for medium intensity free throws, a significant negative correlation exists between the number of fixations on the hoop and the percentage of successful free throws (r = -0.542, P = 0.013). Additionally, there are weak negative correlations observed between number of fixations on backboard (r = -0.186) and net (r = -0.138), and the success rate of free throws. For low-intensity free throws, there was a negligible negative correlation observed between the number of fixations on the backboard (r = -0.081) and the net (r = -0.039), and the percentage of successful free throws. Additionally, a modest positive correlation was found between the number of fixations on the backboard (r = 0.118) and the success rate of free throws. For high-intensity free throws, there was a negligible negative correlation observed between the number of fixations on the hoop (r = -0.012) and the success rate of free throws. Additionally, a weak negative correlation was found between the number of fixations on the backboard (r = -0.145) and the success rate of free throws. Moreover, a moderate negative correlation was identified between the number of fixations on net (r = -0.428) and the success rate of free throws.

**Table 3:** The correlation between number of fixations and the success rate of free throws (N = 20).

	Low intensity		Moderate intensity		High intensity	
AOI	r	p	r	p	r	P
Hoop	-0.081	0.734	-0.542*	0.013	-0.012	0.961
Backboard	0.118	0.612	-0.186	0.431	-0.145	0.541
Net	-0.039	0.869	-0.138	0.562	-0.428	0.060

**Notes**.

* Correlation is significant at the 0.05 level (2-tailed).

The results presented in Table 4 demonstrate a significant positive correlation (r = 0.499, P = 0.021) between the fixation duration on the hoop and the success rate of free throws when the intensity is high. Additionally, there exists a moderate negative correlation (r = 0.335) between the fixation duration the backboard and the success rate of free throws. Furthermore, a weak negative correlation (r = -0.183) was observed between the fixation duration on net and the success rate of free throws. The fixation duration on the hoop exhibited a weak positive correlation (r = 0.220) with the success rate of low-intensity free throws. Moreover, there was a weak negative correlation (r = -0.235) between the fixation duration on the backboard and the success rate of free throws. Furthermore, a moderate negative correlation (r = -0.381) was observed between the fixation duration on the net and the success rate of free throws. The fixation duration of the hoop exhibited a weak positive correlation (r = 0.196) with the success rate of free throws at moderate intensity. Moreover, a moderate negative correlation (r = -0.420) was observed between the fixation duration of the backboard and the success rate of free throws. Additionally, a weak negative correlation (r = -0.117) was identified between the fixation duration of the net and the success rate of free throws.

**Table 4:** The correlation between fixation duration and the success rate of free throws (N = 20).

	Low intensity		Moderate intensity		High intensity	
AOI	r	p	r	p	r	P
Hoop	0.220	0.350	0.196	0.409	0.499*	0.021
Backboard	-0.235	0.319	-0.420	0.065	-0.335	0.138
Net	-0.381	0.098	-0.117	0.624	-0.183	0.428

## Discussion

Fixation serves as the primary means by which participants acquire decision-making information, thereby reflecting their information search strategy. The number of fixations effectively captures the cognitive processing load within the fixation area during free throws, with a higher cognitive load corresponding to an increased number of fixations. Sun emphasized that the ability to regulate both the scope and direction of attention is a crucial component of basketball players’ cognitive capacity, which directly impacts their athletic performance [30]. The average number of fixations of the hoop differed significantly across the three exercise intensities, with longer fixations observed at high intensity. These findings suggest that exercise intensity modulates players’ attentional focus and efficiency during free throw. Concentration and directivity of attention are intricately linked and inseparable, representing two facets of the same cognitive process [20]. Focusing on directivity entails participants selectively attending to specific regions while observing free throws, thereby maintaining mental engagement within a particular area. Fixation concentration not only directs attention towards a specific region but also suppresses irrelevant or potentially disruptive cognitive activity during free throw performance. In low and moderate intensity free throws, players rely on their expertise in theoretical knowledge, automatic motor skills, and accumulated experience to free throw. Additionally, they employ a habitual strategy for information retrieval, the phenomenon is demonstrated as an efficient strategy for information fixation, enabling focused attention and a clear fixation target. It facilitates the rapid identification of the fixation area within the range of the target, extraction of pertinent information from it, and subsequent processing to generate more rational responses. However, as exercise intensity increases, both physical and mental fatigue can impact the players’ habitual information search strategy, leading to alterations in attention concentration and focus. This is evident through an increase in number of fixation and changes in fixation distribution during free throws. The experimental results demonstrated that the hoop accounted for the highest percentage of average number of fixations when players performed free throws at three different exercise intensities. Thus, it can be inferred that the hoop serves as the primary target location for information search during free throw execution. The reason may be attributed to the guidance provided by coaches or teachers during the initial stages of shooting practice, wherein athletes develop a habitual focus on aiming at the basket. This point of aim refers to the specific location on the basket or backboard where their gaze is fixed while shooting [30].

A longer fixation duration at a specific point during the free throws signifies enhanced fixation stability and more refined information processing. Stability is associated with inherent character-ristics of the object itself and its own state. Athletes’ physical fatigue, diminished interest in activities, or weakened willpower pose challenges to maintaining stable attention [20]. Prior to shooting, players are capable of directing their focus towards fixation more effectively, thereby facilitating more refined information processing [3, 31]. The fixation duration exhibited a significant disparity among free throw with varying exercise intensities, with the high-intensity free throw demonstrating the longest average total fixation duration. This finding suggests that exercise intensity negatively impacts information processing efficiency and alters the extent of information processing. The average fixation duration for the moderate intensity free throw was the shortest, with each position exhibiting varying average fixation duration. Conversely, the highest average fixation duration was observed for the hoop in the three intensities free throw, while the average fixation duration for the hoop in the moderate intensity free throw accounted for 52.7% of the total average fixation duration. The findings suggest that the hoop serves as a focal point for information processing during free throws, and moderate-intensity players exhibit high levels of information efficiency and depth. In general, a higher cognitive processing load and longer fixation duration are observed when the cognitive processing load in the fixation range is lower [32]. The change in fixation stability may be attributed to the increased exercise intensity, resulting in higher energy expenditure and accumulation of fatigue among participants, thereby imposing a greater mental load. In order to ensure a successful free throw rate, players strive to concentrate their attention on the target location; however, their ability to control attention is diminished due to the influence of high-intensity exercise load. Simultaneously, as exercise intensity escalates, heart rate elevates and mental stability diminishes. This disrupts the automatic information processing mode of athletes and impairs the control ability of the cognitive system in eye movement regulation, consequently resulting in alterations to fixation stability and an expansion of athletes’ fixation range.

The number of fixations on the hoop exhibited a significant negative correlation with free throw percentage in moderate intensity free throws, while the fixation duration on the hoop showed a significant positive correlation with free throw percentage in high intensity free throws. The findings demonstrate that, within a specific time frame, an increased number of fixations at the hoop during moderate intensity free throws is associated with a lower free throw percentage, while longer fixation durations are linked to higher free throw percentages. These results align with previous research suggesting that longer fixation duration and fewer number of fixations contribute to optimal fixation patterns for enhanced motor performance [33–35]. The free throw percentage of low intensity is not as high as that of moderate intensity, and the free throw percentage of high intensity is the lowest. This may be attributed to the relationship between exercise intensity and players’ cognitive arousal levels, where moderate exercise intensity generates optimal arousal levels conducive to performance, while high exercise intensity inhibits arousal levels (Heart rate > 138 BPM), resulting in poor performance [36–38].

Moreover, research has demonstrated that varying exercise intensities can alter the body’s hormonal composition, thereby impacting cognitive task performance [39]. A combination of psychological and physiological perspectives may elucidate the variances in performance across different exercise intensities: moderate exercise intensity can elicit an optimal level of arousal, while plasma epinephrine and norepinephrine levels tend to escalate at this intensity, with a positive correlation observed between these hormone levels and cognitive performance [39]. Consequently, this leads to enhanced cognitive and motor performance as well as an optimized free throw percentage. However, low intensity fails to attain the optimal arousal level of athletes, while high intensity can impede athletes’ cognitive arousal level, elevate blood ammonia and lactate levels, and compromise both cognition and exercise performance [37].

## Conclusion

Players primarily focus on the hoop position during free throw. The fixation stability is optimal for moderate intensity free throw, exhibiting heightened information search strategies and enhanced information processing efficiency. A longer fixation duration positively correlates with a higher success rate in free throws, while an increased number of fixations negatively impacts the success rate.
